# Inhibitory Control Tells Only Part of the Story: Reward Sensitivity Moderates the Prospective Link Between the No‐Go P3 and Emerging Eating Pathology in Early Adolescents

**DOI:** 10.1002/eat.70172

**Published:** 2026-07-09

**Authors:** Jaron X. Y. Tan, Lisa Brooks, Pan Liu

**Affiliations:** ^1^ Department of Psychology University of Alberta Edmonton Alberta Canada

**Keywords:** disordered eating, early adolescence, event‐related potentials, inhibitory control, reward responsiveness

## Abstract

**Objective:**

Early adolescence is characterized by the onset of mental health problems, including eating pathology (e.g., body image concerns, restrictive eating). Inhibitory control (IC) and reward sensitivity (RS) are two key traits that have been implicated in the development of eating problems. IC is defined as the ability to suppress a prepotent response to facilitate goal‐directed behavior; RS refers to individuals' responsivity to reward‐related stimuli (e.g., food). Despite growing research on IC and RS, little work has examined their joint contribution in predicting emerging eating problems in early adolescents. Further, no work has examined how neurophysiological correlates of IC, like the P3 component, interact with RS to predict subsequent eating pathology.

**Method:**

We analyzed two waves of data from 63 healthy early adolescents (39 girls; Mean age/SD at T1 = 11.01/1.18 years), collected approximately 1 year apart. Youths completed an EEG Go/No‐Go task and reported on their eating problems at T1 and T2; they also reported on their RS at T1.

**Results:**

We found a moderating effect of RS on the association between the No‐Go P3 and disordered eating symptoms: controlling for T1 disordered eating symptoms, a smaller No‐Go P3 at T1 predicted greater disordered eating symptoms at T2, specifically for those with higher RS at T1. Contrary to our expectations, higher, but not lower, commission errors at T1 predicted lower disordered eating symptoms at T2.

**Discussion:**

We contribute novel evidence on the moderating effect of RS on the prospective association between IC and eating pathology in early adolescence. Future studies can leverage these findings to inform IC‐related preventions for youths at risk for eating problems, particularly those with heightened RS.

## Summary

- We found a significant interaction between the inhibition‐related P3 and reward sensitivity in predicting disordered eating symptoms a year later during early adolescence.
- Compared to those with lower reward sensitivity, youths with a smaller P3 who showed higher reward sensitivity at baseline reported greater eating problems a year later.
- Youths who had higher commission errors at baseline reported lower disordered eating symptoms a year later.
- The inhibition‐related P3 and commission error rate may index distinct aspects of inhibitory control.

## 1 Introduction

The transition into adolescence is a critical developmental period characterized by heightened vulnerability to mental health problems, including eating pathology (e.g., body image concerns, restrictive eating; Murray et al. 2022). Such eating problems can predict the later emergence of clinically significant eating disorders (Neumark‐Sztainer et al. 2006) and comorbid mental health conditions (Bulik 2002). Identifying early risk markers during this developmental period is therefore essential for early detection and prevention efforts. Existing research has widely used self‐report and behavioral measures to examine risk factors for eating pathology (Harrison et al. 2010; McClelland et al. 2016). However, these measures may not fully capture early neurocognitive risks that precede observable behavioral deficits and symptoms. Recent work, including our own, has increasingly leveraged neural measures, such as event‐related potentials (ERPs), to examine the neurophysiological markers that may signal early vulnerabilities to eating pathology (Forester et al. 2022; Liu and Tan 2024a, 2024b).

One cognitive risk factor that has received growing attention in relation to eating pathology is inhibitory control (IC), the ability to suppress a prepotent response in order to facilitate goal‐directed behavior (Logan and Cowan 1984). Extant self‐report and behavioral findings have linked poorer IC to greater eating pathology in adults (Wu, Hartmann, et al. 2013; Bartholdy, Dalton, et al. 2016). Both adult women and men with bulimia nervosa showed longer reaction times when inhibiting their responses on a speeded response task relative to healthy controls (Wu, Giel, et al. 2013). Women with anorexia and bulimia nervosa made more errors on a similar response inhibition task compared to healthy controls (Rosval et al. 2006). In a healthy adult sample, overweight adults, compared to normal‐weight adults with low dietary restraint, made more errors on a food‐based response inhibition task (Price et al. 2016).

Emerging, although less consistent, findings have elucidated similar associations between IC and eating problems in children and adolescents (Ramalho et al. 2023). Some studies have reported that poorer IC was associated with greater disordered eating symptoms. Youths aged 14–16 with self‐reported IC problems showed more uncontrolled eating behaviors (Nelson et al. 2020). Among 14‐ to 17‐year‐olds, higher commission errors on a response inhibition task were associated with more binge eating behavior in girls, and greater intake of sweet snacks and beverages in boys (Ames et al. 2014). In a longitudinal study, children with better IC at 10 years old were less likely to have binge eating symptoms when they were 14 years old (Schaumberg et al. 2020). Other studies, however, have found no associations between IC and eating problems in youths (Nelson et al. 2020; Thomas et al. 2024). Youths aged 10–12 with and without disordered eating symptoms showed no differences in IC‐related behavior performance (e.g., inhibition accuracy) on a response inhibition task (Thomas et al. 2024).

To better understand the neural mechanisms of IC and its association with eating pathology, recent ERP studies have examined the neurophysiological correlates of IC (Reyes et al. 2015; Thomas et al. 2024). One such correlate is the inhibition‐related P3 component, a positive deflection peaking approximately 300–600 ms following stimulus onset over frontocentral regions in older children and adults during response inhibition tasks (e.g., stop signals paradigms; Polich 2007). In these tasks, stop signals (i.e., the cue to inhibit responses) typically elicit a larger P3 amplitude than go signals (Wessel and Aron 2015). The P3 is thought to reflect the mobilization of neural resources in executing IC (Cragg et al. 2009), with larger P3 amplitudes indicating more efficient mobilization and potentially more successful inhibition. The inhibition‐related P3 is developmentally sensitive and tends to increase in amplitude and decrease in latency (e.g., peaking earlier) with age (Jonkman 2006; Lewin et al. 2023). For instance, one study in 3‐ to 10‐year‐olds found that the inhibition‐related P3 peaked over a relatively later and broader 600–900 ms time window (Lewin et al. 2023). Studies have also found a developmental shift in the topography of the P3, which gradually moves away from parietal regions and toward frontocentral regions beginning in middle childhood (~6 years of age) and continuing throughout adolescence (Jonkman 2006; Paitel and Nielson 2021; Wessel and Aron 2015). These changes in the P3 may reflect more efficient IC as cognitive control processes and the prefrontal cortex mature over time (Abdul Rahman et al. 2017; Johnstone et al. 2007).

Increasing work has linked the inhibition‐related P3 to eating problems across development (Andreu et al. 2024; Reyes et al. 2015; Walk et al. 2020; Yue et al. 2020). In adults, individuals with anorexia nervosa exhibited smaller inhibition‐related P3 amplitudes compared to healthy controls (Yue et al. 2020). Among 9‐ to 10‐year‐old youths, those who were overweight or obese showed smaller P3 compared to their normal‐weight peers (Reyes et al. 2015; Walk et al. 2020). However, one study in 10‐ to 12‐year‐olds found no associations between the P3 and disordered eating (Thomas et al. 2024). Further, extant studies on the relationship between the inhibition‐related P3 and eating pathology were cross‐sectional; longitudinal work is needed to examine the prospective associations between the inhibition‐related P3 and disordered eating, particularly during early adolescence.

The inconsistent findings in the associations between IC and eating pathology may be partially related to the fact that these studies did not account for other factors that may interact with IC in predicting the eating pathology outcomes. The developmental psychopathology perspective contends that it is not a single factor, but rather the interplay among multiple factors, that contributes to the development of psychopathological outcomes (Cicchetti and Toth 2009). Consistent with this perspective, dual‐process theories posit that goal‐directed behaviors are shaped by the interplay between multiple distinct yet complementary systems. In particular, higher‐order regulatory processes, such as IC, interact with lower‐order, reactive processes to influence behavioral and emotional responses to the environment (Derryberry and Rothbart 1997; Eisenberg et al. 1994). For example, community‐dwelling youths with lower executive control and higher negative affect at age seven showed steeper increases in depressive symptoms from age seven to age nine compared to their peers with lower control but lower negative affect (Kotelnikova et al. 2015). Similar interactions between higher‐order regulatory and lower‐order reactive processes may contribute to eating pathology risks.

One lower‐order reactive process that may be particularly relevant to eating pathology is reward sensitivity (RS), a trait‐like construct defined as individuals' responsivity to reward‐related stimuli (e.g., food; Loxton 2018). Heightened RS has been linked to increased perceived salience of food cues and greater vulnerability to maladaptive eating behaviors in adults and youths (Harrison et al. 2010). In a cross‐sectional study, overweight adults with binge eating disorder showed greater activation in reward‐related brain regions (e.g., medial orbitofrontal cortex) in response to high‐calorie food images than overweight adults without binge eating disorder and normal weight controls (Schienle et al. 2009). In addition to food‐specific RS, more domain‐general RS, including sensitivity toward other types of reward (e.g., money), also plays a role in the emergence of disordered eating behaviors (Ramalho et al. 2023). Young adults with higher self‐reported reward drive in general showed greater restrictive eating tendencies (Jonker et al. 2021). Among children with obesity, those who were prone to binge eating behaviors showed greater RS to monetary reward than those who did not struggle with binge eating (Nederkoorn et al. 2006). Higher activation in the ventromedial prefrontal cortex (another brain region supporting reward processing) in response to monetary reward was correlated with more severe binge eating in female adolescents (Bodell et al. 2018). This suggests a general association between RS and eating pathology, regardless of the specific reward stimuli.

Informed by the dual‐process framework, a few studies have further examined the interaction between IC and RS in relation to eating pathology, including restrictive eating patterns (Jonker et al. 2018; Matton et al. 2017; Munsch et al. 2019; Van Malderen et al. 2018). In adults and adolescents, higher RS was associated with more restrained eating only for those with lower IC, as indicated by slower responses on incongruent trials (Jonker et al. 2018) or greater difficulty suppressing automatic responses in incongruent trials (Matton et al. 2017) on a Stroop task. Consistent with the dual‐process perspective, these findings suggest that youths with higher RS may not be at risk for eating problems if they are able to inhibit and properly regulate problematic eating‐related cognitions and behaviors, including dietary restraint and body‐related concerns. Likewise, lower IC in youths may not necessarily predict greater disordered eating when youths are not highly sensitive to reward.

Despite the growing research on IC, RS, and their interaction in relation to eating pathology, the literature has several notable gaps. Most studies have focused on adults, with fewer examining these relations in children and adolescents (Matton et al. 2017; Munsch et al. 2019; Van Malderen et al. 2018). Besides that, the majority of existing work in this area has relied on cross‐sectional designs and self‐report or behavioral measures of IC, which limits our understanding of the neurocognitive substrates of IC and their role in predicting eating pathology over time. No work has examined to what extent the neurophysiological correlates of IC, such as the inhibition‐related P3, interact with individual differences in RS to predict prospective symptoms of disordered eating during early adolescence. Early adolescence is characterized by significant developmental changes in both IC and reward processes. In particular, lower‐order reactive processes involved in RS tend to mature earlier than higher‐order regulatory processes supporting IC, potentially resulting in an imbalance between heightened RS and still‐maturing IC capacities (Cantou et al. 2025; Steinberg 2010). Given these developmental changes and the emergence of eating problems during early adolescence, this period provides a unique window to examine the early risk processes of eating pathology.

Using two‐wave longitudinal data collected from a community sample of 63 9‐ to 12‐year‐old youths, the present study investigated the prospective associations between the behavioral (e.g., commission error rate) and neural (e.g., P3) measures of IC at baseline and disordered eating symptoms a year later. We also examined to what extent youths' self‐reported RS at baseline moderated these associations (or interacted with IC in predicting eating problems). Specifically, we focused on domain‐general RS to capture the broader vulnerability toward eating pathology that may precede alterations in food‐specific RS and the emergence of clinically significant eating disorders (Loxton 2018; Maxwell et al. 2017; Schreuders et al. 2018). Based on the existing literature, we hypothesized that (1) a smaller inhibition‐related P3 and higher commission error rate would predict greater symptoms of disordered eating a year later, with baseline symptoms controlled for, and (2) higher RS at baseline would exacerbate the prospective associations between the P3/commission error rate and disordered eating.

## 2 Method

### 2.1 Participants and Procedure

Data were drawn from a larger longitudinal project examining cognitive mechanisms associated with risk for psychopathology during late childhood and early adolescence. At Time 1 (T1), 115 unselected community‐dwelling youths (66 girls; Mean age/SD = 10.99/1.18 years) and their caregivers from a Midwestern urban region in the United States completed an in‐person laboratory assessment. Sample demographics were broadly consistent with those of the surrounding community: 87.5% identified as White, 3.6% as Asian, and 8.9% as multiracial; 7.2% identified as Hispanic or Latino. Annual family income ranged from $15,000 to $350,000.

Caregivers provided written informed consent and youths provided assent prior to participation. Youths then completed four EEG tasks, including a Go/No‐Go task, presented in counterbalanced order, as well as an eye‐tracking task assessing multiple cognitive biases. After the laboratory session, youths completed self‐report questionnaires administered through Qualtrics assessing socioemotional functions and psychopathology symptoms, including RS and disordered eating. Participants returned approximately 12 months later for a follow‐up assessment (Time 2; T2). At T2, 92 youths (53 girls; Mean age/SD = 12.06/1.20 years) completed the same questionnaire battery and laboratory tasks administered at T1. Participants received monetary compensation after completing each assessment wave. All procedures were approved by the Institutional Review Board. The present analyses focused on the EEG and behavioral data of the Go/No‐Go task and the self‐report data of RS at T1 and disordered eating symptoms at T1 and T2.

### 2.2 Self‐Report Measures

Reward sensitivity (RS) was assessed at T1 using the Behavioral Activation System (BAS) scale, a 13‐item self‐report measure of individual differences in reward responsiveness, behavioral drive, and fun‐seeking tendencies (Carver and White 1994). Each item (e.g., “When I get something I want, I feel excited and energized”) was rated on a four‐point Likert scale ranging from 1 (*very false for me*) to 4 (*very true for me*). A total BAS score was calculated (Range = 13–52) as an indicator of RS. The BAS scale demonstrated good internal consistency in the current sample (Cronbach's *α* = 0.78).

Disordered eating behaviors were assessed using the Child Eating Disorder Examination Questionnaire–Short Form (ChEDE‐Q8; Kliem et al. 2017), an eight‐item self‐report measure that assesses eating problems (e.g., restrictive eating, concerns about body weight and shape) in youths aged 7–18 years. For each item, participants indicated how often the statement applied to them during the previous 2 weeks using a seven‐point scale ranging from 0 (*no days*) to 6 (*every day*; e.g., “Have you felt fat?”). Item responses were summed to create a total score ranging from 0 to 48, with higher scores indicating greater disordered eating behaviors. Internal consistency was high in the present sample (Cronbach's *α* at T1 = 0.92; *α* at T2 = 0.93).

Body mass index percentiles (BMI%) at T1 were included as a covariate in the analysis (Calzo et al. 2012). BMI was calculated using participants' self‐reported height and weight (BMI = kg/m^2^). BMI values were then converted to BMI% based on age‐ and sex‐specific normative data from the US Centers for Disease Control and Prevention (Centers for Disease Control and Prevention 2022). BMI% represents a youth's relative standing compared to peers of the same age and sex in the general population.

### 2.3 The EEG Go/No‐Go Task

Participants completed a visual Go/No‐Go task while EEG was recorded (Falkenstein et al. 1999; Grammer et al. 2014). The task included 315 trials consisting of 252 Go trials and 63 No‐Go trials, distributed across three blocks. Each block contained 84 Go trials and 21 No‐Go trials, and trials were presented in randomized order within each block. Each trial began with a fixation cross displayed for 200 ms, followed by the presentation of a black‐and‐white image for 300 ms depicting either a cat or a dog. Participants were instructed to press a response button as quickly as possible when a cat appeared (Go trial) and to withhold responding when a dog appeared (No‐Go trial). Following the button press (or the 300 ms image with no response), a second fixation cross was presented for 900 ms before the onset of the next trial. The task was administered using E‐Prime software (Psychology Software Tools Inc., Pittsburgh, PA). Prior to the experimental blocks, participants completed a practice session to ensure familiarity with the task instructions. Participants' commission error rate (i.e., the proportion of No‐Go trials on which a response occurred) was used as the behavioral index of IC.

### 2.4 EEG Data Acquisition and Processing

Participants completed the Go/No‐Go task in an electrically shielded chamber while continuous EEG was recorded using a 64‐channel HydroCel Geodesic Sensor Net (Electrical Geodesics Inc.) and a NetAmps 200 amplifier. EEG signals were sampled at 250 Hz and recorded online with reference to the vertex electrode (Cz). Electrode impedances were maintained below 50 kΩ during data collection. EEG preprocessing was conducted using the EEGLAB (Delorme and Makeig 2004) and ERPLAB (Lopez‐Calderon and Luck 2014) toolboxes in MATLAB (MathWorks Inc., Natick, MA). Continuous EEG data were first bandpass filtered between 0.1 and 40 Hz and then re‐referenced to the average of the left and right mastoid electrodes. Ocular artifacts, including eye blinks and eye movements, were corrected using an independent component analysis (ICA) procedure.

EEG data were time‐locked to stimulus onset and segmented into epochs extending from 200 ms prior to stimulus onset to 1000 ms following stimulus onset. Epochs were baseline corrected using the 200 ms pre‐stimulus interval. Additional artifact rejection criteria were applied to remove contaminated trials, including (1) voltage fluctuations exceeding ±100 μV, (2) voltage changes greater than 50 μV between adjacent time points, and (3) peak‐to‐peak voltage differences exceeding 300 μV within a 200 ms moving window. Trials were sorted by Go and No‐Go condition, and only correct‐response trials were included in ERP averaging. Participants were retained for analysis if they had at least 10 artifact‐free trials per condition (Olvet and Hajcak 2009), resulting in a sample of 102 participants (62 girls; Mean age/SD = 10.99/1.15 years).

### 2.5 ERP Quantification

Prior research has indicated a developmental shift in the No‐Go P3, such that the P3 peaks earlier (Jonkman 2006) and moves from parietal regions to more frontocentral regions with age (Wessel and Aron 2015). Consistent with this, visual inspection of the grand average waveforms in the current study showed a frontocentral P3, with a later time window that aligns with studies in younger youth samples (e.g., 3‐ to 10‐year‐olds; Jonkman 2006; Lewin et al. 2023). Notably, a stronger parietal P3 remains observable in our data, which is also consistent with previous work in children (Abdul Rahman et al. 2017; Spronk et al. 2008) and likely reflects youths' continued recruitment of more general attentional processes to support response inhibition during the task (e.g., stimulus differentiation; Jonkman 2006; Paitel and Nielson 2021). Based on both the visual inspection and prior literature, we focused on the frontocentral P3 as an indicator of IC and quantified it as the mean amplitude of the 500–800 ms post‐stimulus time window across six frontocentral electrodes (F1, Fz, F2, FC1, FCz, FC2). Paired‐samples *t*‐test indicated that the No‐Go P3 amplitude (Mean/SD = 2.54/4.43) was significantly larger than the Go P3 amplitude, (Mean/SD = 0.96/1.80, *t*(101) = 7.30, *p* < 0.001). Figure 1 shows the grand average waveforms and topographic maps of the P3 component at FCz for the Go Correct trials, No‐Go Correct trials, and the difference wave between the two (No‐Go Correct minus Go Correct).

**FIGURE 1:**
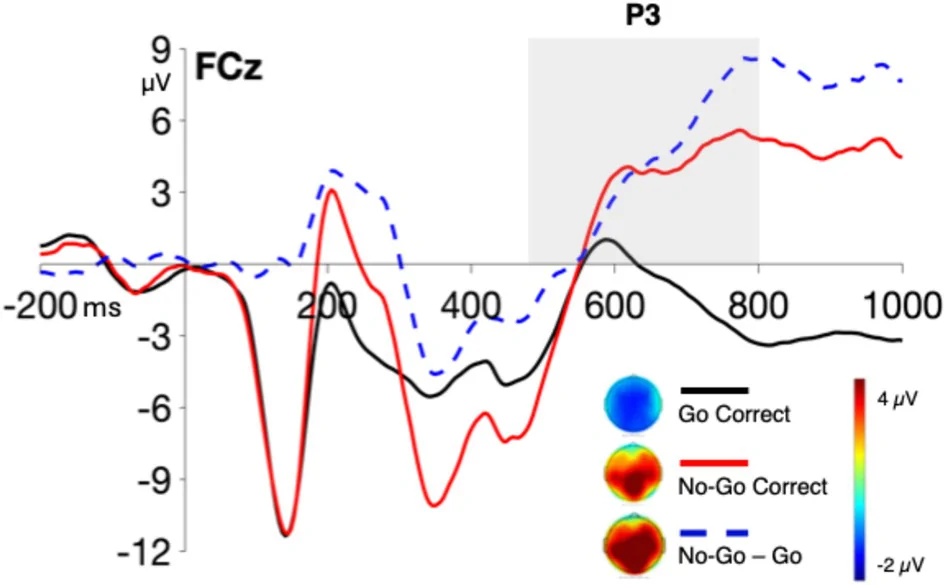
Grand average ERP waveforms and topographic maps of the P3 component at FCz for Go Correct trials, No‐Go Correct trials, and the difference wave between the two (No‐Go Correct minus Go Correct). μV, microvolts; ms, milliseconds.

To isolate the inhibition‐specific neural activity, we used a regression‐based residualization approach by regressing the No‐Go P3 amplitude on the Go P3 amplitude and extracted the standardized residuals to index the P3 (Meyer et al. 2017). These residualized values represented variance in the No‐Go P3 that was independent of general task‐related P3 activity, providing a more accurate measure of IC‐related processes.

### 2.6 Statistical Analyses

Of the 115 youths at T1, 31 had missing data for at least one study variable (e.g., eight with missing disordered eating symptoms, 13 with unusable ERP data, and 21 with missing BMI%). At T2, seven out of the 92 youths that returned had missing data for disordered eating symptoms. Little's Missing Completely at Random Test (Little 1988) using the R *naniar* package (Tierney and Cook 2023) indicated that the data were missing completely at random (*χ* ^2^(362) = 390.00, *p* = 0.153).^1^ Participants with complete data at T1 and T2 (*n* = 63; 39 girls; Mean age/SD at T1 = 11.01/1.18 years) were retained, and their data were subjected to subsequent statistical analysis in R Studio (Version 2023.03).^2^

We conducted two hierarchical multiple regressions to examine the moderating effect of T1 RS on the associations between the T1 residualized P3/commission error rate and T2 disordered eating symptoms. In both models, T2 disordered eating was treated as the outcome. In Step 1, T1 disordered eating and T1 BMI% were entered as covariates in both models. Despite documented sex differences in disordered eating behaviors (Breton et al. 2023) and age‐related changes in the inhibition‐related P3 (Jonkman 2006), we did not include these variables as covariates in our main models due to our modest sample size (*n* = 63) in statistical analyses.^3^ In Step 2, T1 residualized P3 or commission error rate was added as predictors in each model, along with T1 RS. In Step 3, the residualized P3 × RS and the commission error rate × RS interaction terms were added to each model, respectively.

## 3 Results

### 3.1 Descriptive Statistics and Bivariate Correlations Between Study Variables

Table 1 shows the means, standard deviations, and bivariate correlations of the study variables. Higher BMI% at T1 was associated with greater disordered eating at both T1 and T2. Disordered eating symptoms at T1 and T2 were positively correlated with each other. Independent samples *t*‐tests indicated significant sex differences in the T1 No‐Go P3, T1 residualized P3, and T1 commission error rate. Compared to boys, girls showed a smaller T1 No‐Go P3 (*t*(76) = 2.05, *p* = 0.044; girls: Mean/SD = 1.98/4.51; boys: Mean/SD = 4.18/4.89), a smaller T1 residualized P3 (*t*(76) = 2.05, *p* = 0.044; girls: Mean/SD = −0.90/4.49; boys: Mean/SD = 1.30/4.91). Girls and boys did not differ in commission error rate at T1 (*p* = 0.144).

**TABLE 1:** Mean, standard deviation, and bivariate correlations of the study variables.

	1	2	3	4	5	6	7	8	9
1. Age of years at T1									
2. T1 BMI%	−0.15								
3. T1 Go P3	0.01	0.09							
4. T1 No‐Go P3	0.06	−0.20	−0.04						
5. T1 Residualized P3	0.01	−0.17	0.00	1.00**					
6. T1 Error Rate	−0.02	0.08	0.05	0.04	0.05				
7. T1 BAS Scores	−0.09	0.16	0.13	0.07	0.06	0.02			
8. T1 Disordered Eating	0.12	0.34**	0.03	0.05	0.04	−0.08	0.12		
9. T2 Disordered Eating	0.11	0.34**	0.10	−0.04	−0.03	−0.20	0.12	0.56**	
Mean	10.99	57.83	−0.96	2.54	0.00	0.42	39.00	5.77	6.24
SD	1.18	32.57	1.80	4.43	4.76	0.18	5.33	10.45	10.54

*Note:* *p < 0.05, **p < 0.01 (2‐tailed). Means and standard deviations for age are based on the full sample (*N* = 115).

Abbreviations: BAS, Behavioral Activation System; BMI%, body mass index percentile; SD, standard deviation.

### 3.2 Results of Hierarchical Regression Analysis

Tables 2 and 3 present the results of the hierarchical multiple regression analysis with residualized P3 and commission error rate as the predictor, respectively. As shown in Table 2, in the covariate only model (Step 1), T1 disordered eating symptoms predicted T2 disordered eating symptoms (*β* = 0.31, SE = 0.11, *p* = 0.014). Controlling for T1 symptoms and BMI% (Step 2), we found a marginal main effect of T1 residualized P3 (*β* = −0.23, SE = 0.23, *p* = 0.053), with a larger residualized P3 at T1 marginally associated with lower disordered eating symptoms at T2. More interestingly, there was a significant residualized P3 × RS interaction (Step 3; *β* = −0.26, SE = 0.04, *p* = 0.029).^4^ Decomposing the interaction showed that a smaller residualized P3 predicted greater disordered eating symptoms for youths with higher (mean + 1SD; *B* = −0.85, SE = 0.28, *p* = 0.004), but not mean (*B* = −0.41, SE = 0.22, *p* = 0.071) or lower (mean − 1SD; *B* = 0.04, SE = 0.31, *p* = 0.895) RS (see Figure 2).

**TABLE 2:** Results of hierarchical regression with T1 residualized P3 and T1 reward sensitivity predicting T2 disordered eating.

		*B*	*β*	SE	*t*	*p*	Adjusted *R* ^2^
Step 1: Covariates only	T1 Disordered Eating	0.29	0.31	0.11	2.53	0.014*	0.16
	T1 BMI%	0.06	0.22	0.03	1.84	0.071	
Step 2: Main effects	T1 Residualized P3	−0.45	−0.23	0.23	−1.98	0.053	0.20
	T1 Reward Sensitivity	0.33	0.18	0.21	1.56	0.124	
	T1 Disordered Eating	0.31	0.34	0.11	2.81	0.007**	
	T1 BMI%	0.04	0.14	0.03	1.15	0.255	
Step 3: Interaction effects	T1 Residualized P3	−0.40	−0.21	0.22	−1.84	0.071	0.25
	T1 Reward Sensitivity	0.37	0.21	0.21	1.82	0.074	
	T1 Disordered Eating	0.30	0.32	0.11	2.72	0.009**	
	T1 BMI%	0.02	0.09	0.03	0.73	0.469	
	P3 × Reward Sensitivity	−0.09	−0.26	0.04	−2.24	0.029*	

*Note:* *p < 0.05, **p < 0.01.

Abbreviations: BMI%, body mass index percentile; SE, standard error; T1, Time 1; T2, Time 2.

**TABLE 3:** Results of hierarchical regression with T1 error rate and T1 reward sensitivity predicting T2 disordered eating.

		*B*	*β*	SE	*t*	*p*	Adjusted *R* ^2^
Step 1: Covariates only	T1 Disordered Eating	0.29	0.31	0.11	2.53	0.014*	0.16
	T1 BMI%	0.06	0.22	0.03	1.84	0.071	
Step 2: Main effects	T1 Error Rate	−12.58	−0.23	6.22	−2.02	0.048*	0.20
	T1 Reward Sensitivity	0.30	0.17	0.21	1.44	0.157	
	T1 Disordered Eating	0.25	0.27	0.11	2.25	0.028*	
	T1 BMI%	0.06	0.21	0.03	1.72	0.091	
Step 3: Interaction effects	T1 Error Rate	−11.77	−0.22	6.22	−1.89	0.064	0.21
	T1 Reward Sensitivity	0.33	0.18	0.21	1.57	0.123	
	T1 Disordered Eating	0.25	0.27	0.11	2.25	0.028*	
	T1 BMI%	0.05	0.18	0.03	1.51	0.137	
	Error Rate × Reward Sensitivity	−1.60	−0.14	1.30	−1.23	0.223	

*Note:* *p < 0.05.

Abbreviations: BMI%, body mass index percentile; SE, standard error; T1, Time 1; T2, Time 2.

**FIGURE 2:**
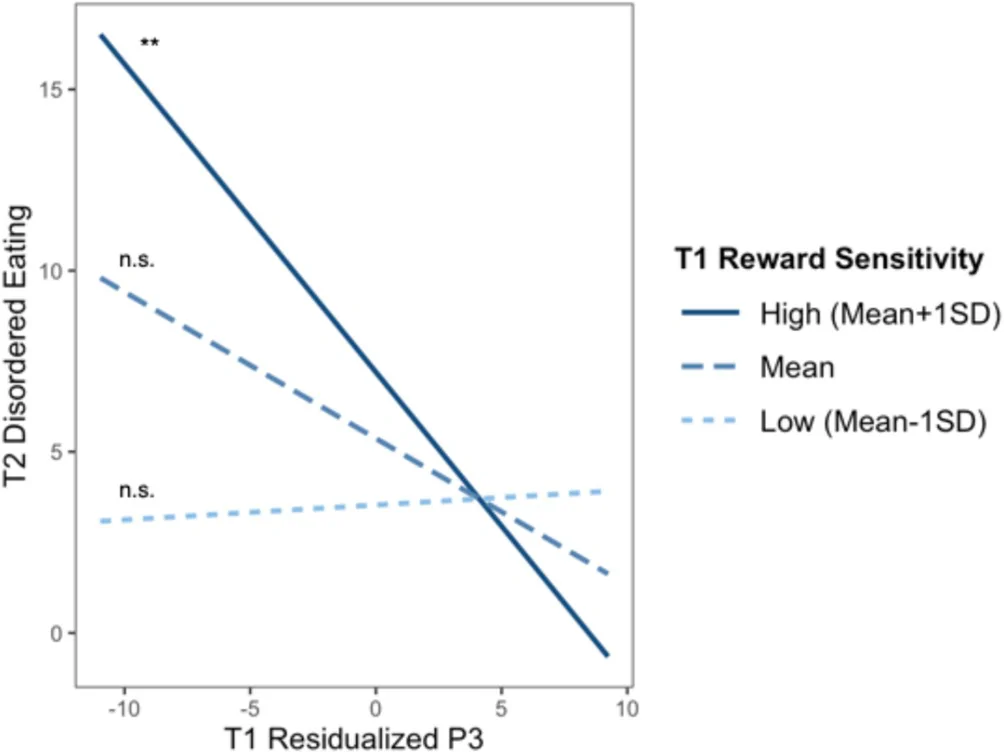
An illustration of the interaction between the T1 residualized P3 and T1 reward sensitivity predicting T2 disordered eating. **p < 0.01; n.s., non‐significant; SD, standard deviation; T1, Time 1; T2, Time 2.

With commission error rate as the predictor (Table 3), we found a significant main effect of T1 error rate (Step 2; *β* = −0.23, SE = 6.22, *p* = 0.048); greater error commissions at T1 predicted lower disordered eating symptoms at T2, with T1 symptoms controlled for. There was no Error Rate × RS interaction (Step 3; *β* = −0.14, SE = 1.30, *p* = 0.223).

## 4 Discussion

Early adolescence is a developmental period marked by rapid neurocognitive maturation and heightened vulnerability to eating psychopathology. To better understand the etiology of early eating pathology, we for the first time examined the moderating effect of RS on the prospective association between IC (indicated by inhibition‐related P3 and commission error rate) and disordered eating symptoms in a sample of community‐dwelling 9‐ to 12‐year‐old youths. As expected, we observed a significant interaction between the inhibition‐related P3 and self‐reported RS at T1 in predicting disordered eating symptoms a year later, with baseline symptoms controlled for. Specifically, T1 P3 predicted T2 disordered eating symptoms for youths with higher, but not lower RS. Contrary to our expectations, lower commission error rate at T1 was associated with higher symptoms at T2 but did not interact with RS in predicting T2 symptoms. These findings provide novel evidence on the joint contribution of the neural correlates of IC and RS in predicting eating pathology during early adolescence.

Youths with a smaller P3 at T1 reported greater disordered eating symptoms at T2, but only among those reporting higher RS at T1. The magnitude of the inhibition‐related P3 elicited during the Go/No‐Go task (or other stop signal paradigms) has been considered an indicator of the recruitment and mobilization of neural resources in support of IC (Johnstone et al. 2007; Wessel and Aron 2015). A smaller inhibition‐related P3 may reflect inefficient mobilization of neural resources toward inhibiting dominant but goal‐irrelevant responses, which has been associated with poorer developmental outcomes including eating problems (Reyes et al. 2015; Walk et al. 2020). However, some studies have reported a larger inhibition‐related P3 in association with negative outcomes, especially in the context of environmental adversity (Cui et al. 2021; Shackman et al. 2007). In a cross‐sectional study, children aged 11 who were exposed to physical abuse showed a larger P3 in an auditory response inhibition task, which was further associated with greater externalizing behaviors (Cui et al. 2021). This pattern may reflect increased recruitment of cognitive control resources that stemmed from heightened environmental demands and stress exposure over time (McLaughlin and Sheridan 2016; Paitel and Nielson 2021).

In our healthy, low‐risk youth sample, a smaller P3 was predictive of disordered eating symptoms, but only in those who were highly sensitive to reward. Youths with higher RS may experience stronger motivational drives toward rewarding stimuli, such as palatable food. When these youths struggle to efficiently mobilize neurocognitive resources in executing IC, as reflected by a smaller P3, they might not be able to successfully inhibit problematic eating‐related thoughts and behaviors, such as dietary restraint and body‐related concerns. As a result, they might be at increased risk for disordered eating, especially restrictive eating problems (Galimberti et al. 2012; Jonker et al. 2018). In contrast, youths with a smaller P3 but lower RS might experience lower impulses toward reward (including food reward), reducing disordered eating risk associated with inefficient mobilization of resources supporting IC. These findings align with the dual‐process theory (Eisenberg et al. 1994; Steinberg 2010), suggesting that eating pathology may emerge when heightened, lower‐order reactive processes co‐occur with weaker, higher‐order regulatory processes. However, given the smaller sample size in our regression models (*n* = 63), and that the P3 × RS interaction was no longer significant after accounting for age and sex, these findings remain preliminary and require replication in larger samples to confirm the observed effects.

Importantly, the P3 × RS interaction was observed for the frontocentral, but not parietal, P3. As noted earlier, both frontocentral and parietal P3 activity are commonly observed in children during response inhibition tasks; the frontocentral P3 has been closely linked to IC processes, whereas the parietal P3 may reflect the recruitment of broader attentional processes that support response inhibition (Jonkman 2006; Paitel and Nielson 2021). This pattern suggests that RS may be more strongly related to the IC processes indexed by the frontocentral P3. However, given the potential functional overlap between the frontocentral and parietal P3, additional work is needed to elucidate the specific neurocognitive processes underlying these associations.

We also observed a main effect of commission error rate in predicting prospective disordered eating symptoms. However, contrary to our expectations, youths with higher commission error rates at T1 reported lower, rather than higher, disordered eating symptoms at T2. As mentioned earlier, existing behavioral literature on the association between IC and disordered eating in youth has been mixed. Some studies found that higher commission errors in response inhibition tasks were associated with risk for obesity and more eating problems in adolescents (Ames et al. 2014; Ramalho et al. 2023). Others found no associations between behavioral performance of IC and eating problems in adolescents (Andreu et al. 2024; Bartholdy et al. 2019; Walk et al. 2020).

Previous work has suggested that the inhibition‐related P3 and commission error rate may index distinct aspects of IC (Brydges et al. 2014). Indeed, similar to previous work, we found no correlation between the inhibition‐related P3 and commission error rate in this study (Thomas et al. 2024). While the P3 reflects the neural processes preceding a behavioral response, commission error rate directly reflects the success or failure of behavioral responses and may tap into a wider range of cognitive and behavioral processes that contribute to task performance (Thomas et al. 2024; Xia et al. 2020). For example, variability in attention response strategies (e.g., speed‐accuracy trade‐offs) can influence the likelihood of successful response inhibition (Endrass et al. 2012; Tan and Liu 2025; Tompson et al. 2020).

We speculated that in our youth sample, lower commission errors might have partially reflected participants' increased use of preparatory or anticipatory IC strategies. Compared to reactive IC which involves withholding a response in reaction to a cue, anticipatory IC refers to the proactive engagement of IC processes to prepare for inhibitory demands (e.g., by paying closer attention to stimulus features; Criaud et al. 2012; Shen et al. 2023). Anticipatory IC may reflect individuals' intolerance of uncertainty (Bartholdy, Campbell, et al. 2016), which has been found to contribute to over‐controlling eating behaviors such as dietary restrictions (Bartholdy, Campbell, et al. 2016; Shen et al. 2023). This literature might explain the observed association in our data between lower commission error rate and higher ChEDE‐Q8 score, which primarily assess restrictive eating rather than impulsive binge eating behaviors. Indeed, women with anorexia nervosa have been found to show greater anticipatory IC and greater intolerance of uncertainty compared to healthy women and women with binge‐eating disorder (Bartholdy et al. 2017). However, we did not measure anticipatory IC or intolerance of uncertainty in this study. Future work directly examining these constructs and their associations with different types of eating problems (e.g., restrictive vs. impulsive eating tendencies) is needed to clarify the mechanisms through which different types of IC may relate to eating pathology.

Further, we did not find an interaction between T1 commission error rate and RS in predicting disordered eating at T2. Considering the potential differences between the neural and behavioral measures of IC, RS may be uniquely associated with the inhibition‐related P3 in the context of restrictive eating pathology, whereas the behavioral measure of IC may interact with factors other than RS (Bartholdy, Dalton, et al. 2016). Similarly, more work is needed to confirm this speculation.

Our study had several strengths. By assessing disordered eating across two time points over a 12‐month period, we were able to control for baseline disordered eating symptoms and better speak to the directional relationship between T1 P3 and T2 symptoms. We took a multi‐method approach by incorporating behavioral, ERP, and questionnaire measures to examine the associations between constructs, which reduced shared measurement error and allowed for a more comprehensive understanding of the early risk processes for eating pathology. Finally, instead of group comparisons (e.g., between clinical and non‐clinical samples), we took a dimensional approach, examining an unselected community‐dwelling youth sample with emerging eating problems. Such an approach increases statistical power and sheds light on the processes shared by both typical and atypical development (Cicchetti and Toth 2009).

One limitation of this study was the use of a brief, global self‐report measure of eating pathology (e.g., ChEDE‐Q8), which did not differentiate among specific subtypes of disordered eating, such as bulimia nervosa or binge‐eating disorder (Kliem et al. 2017). Although the ChEDE‐Q8 has demonstrated a clear unidimensional factor structure supporting the use of a global score (Kliem et al. 2017), the items primarily assessed restrictive eating behaviors and concerns related to body weight, body shape, and eating, with no direct measure of impulsive eating or binging/purging behaviors. The relatively modest sample size for the moderation analyses (*n* = 63) represents another limitation, as it might have reduced the statistical power to detect interaction effects (e.g., Error Rate × RS) and the stability of the observed effects. Our independent‐samples *t*‐tests and Chi‐square test indicated no significant differences between retained and excluded participants on key study variables; however, the attrition from T1 to T2 might have also affected the stability and generalizability of the observed effects (e.g., due to other unmeasured variables). Future work using larger samples and examining distinct subtypes of disordered eating in high‐risk or clinical youths is needed to better clarify the IC‐disordered eating relations, as IC may have different implications for more overcontrolled, restrictive eating versus more emotionally driven binge‐eating problems (Haynos et al. 2021).

Additionally, the use of a Go/No‐Go task with neutral stimuli in our study limits the interpretability of our findings, as IC of neutral stimuli may not fully generalize to food‐relevant stimuli or contexts, which are more motivationally salient for those with eating‐related problems (Shen et al. 2023). Future work using food‐specific stimuli in a Go/No‐Go task will better capture the IC processes that are more directly relevant to food contexts and eating pathology. In calculating youths' BMI%, we relied on their self‐reported height and weight, which might be prone to reporting biases. Nonetheless, studies have often demonstrated high correlation between self‐reported and actual assessments of height and weight (Rios‐Leyvraz et al. 2022). Finally, the current findings were drawn from a primarily White youth sample from middle‐class families. Future work with marginalized racial/ethnic samples and lower‐income families will be important to evaluate the generalizability of these findings to other populations.

In sum, our study provided novel evidence on the interaction between IC and RS in predicting the development of disordered eating during early adolescence. Specifically, a smaller inhibition‐related P3 predicted greater disordered eating a year later, but only among youths with higher RS. This suggests that early alterations in the inhibition‐related P3 may confer risk for eating pathology particularly in the context of heightened RS. We also highlighted differential associations between behavioral and neural measures of IC and disordered eating symptoms, indicating that these measures may capture distinct facets of IC processes. Together, these findings will inform future intervention research aimed at modifying atypical IC or RS processes and promoting adaptive regulatory strategies in youths at risk for eating pathology.

## Author Contributions

**Jaron X. Y. Tan:** conceptualization, investigation, writing – original draft, writing – review and editing, methodology, formal analysis, data curation, project administration, visualization. **Lisa Brooks:** writing – review and editing, writing – original draft, formal analysis, data curation. **Pan Liu:** funding acquisition, writing – original draft, writing – review and editing, conceptualization, supervision, project administration, methodology, resources.

## Lived Experience Involvement Statement

No specific efforts were undertaken to involve persons with lived experience in the study design or execution, or in the preparation of this manuscript.

## Funding

The current study was supported by a NIGMS Centers of Biomedical Research Excellence (P30 GM114748, “COBRE Center for Visual and Cognitive Neuroscience”) pilot grant to Pan Liu and a Graduate Studentship Award from the Women and Children's Health Research Institute to Jaron X. Y. Tan. This study was not preregistered.

## Conflicts of Interest

The authors declare no conflicts of interest.

## Data Availability

The data that support the findings of this study are available from the corresponding author, Jaron X. Y. Tan, upon reasonable request due to privacy and ethical restrictions.

## Endnotes

^1^
Independent‐samples *t*‐tests and a Chi‐square test indicated that participants retained in the study did not differ significantly from those excluded due to missing data on T1 BMI%, *t*(93) = 1.96, *p* = 0.053; T1 disordered eating symptoms, *t*(105) = 1.19, *p* = 0.236; T2 disordered eating symptoms, *t*(83) = 1.43, *p* = 0.156; age, *t*(113) = −0.19, *p* = 0.846; or sex, *χ* ^2^(1) = 0.79, *p* = 0.375.

^2^
Sensitivity analyses with the imputed data (*N* = 115) demonstrated similar patterns of associations for the residualized P3 and commission error rate, although the effects were smaller. The main effect of the T1 residualized P3 was not significant (*β* = 0.01, SE = 0.20, *p* = 0.894); however, there was a marginal T1 P3 ⋅ RS interaction effect (*β* = −0.15, SE = 0.03, *p* = 0.069). There was a significant main effect of T1 error rate (*β* = −0.20, SE = 4.56, *p* = 0.015).

^3^
Given the observed sex differences in T1 residualized P3 and the potential age differences across study variables, we conducted sensitivity analyses by controlling for sex and age as covariates in the regression models. The overall pattern of results remained similar after including these covariates. Specifically, there was a marginal T1 residualized P3 × RS interaction effect (*β* = −0.24, SE = 0.04, *p* = 0.053) and a marginal main effect of T1 error rate (*β* = −0.21, SE = 6.31, *p* = 0.079). There was no significant main effect of T1 residualized P3 (*β* = −0.20, SE = 0.24, *p* = 0.113).

^4^
We conducted similar regression analyses on the T1 parietal P3, which was quantified as the residualized difference score (No‐Go minus Go Correct) of the mean amplitude within the 400–600 ms post‐stimulus time window across six parietal electrodes (P1, P2, Pz, POz, PO3, PO4). Paired‐samples *t*‐test indicated that the parietal No‐Go P3 amplitude (Mean/SD = 4.99/4.02) was significantly larger than the parietal Go P3 amplitude (Mean/SD = 0.63/1.82, *t*(101) = 10.35, *p* < 0.001). Controlling for T1 BMI% and disordered eating symptoms, there was a marginal main effect of T1 parietal P3 (*β* = −0.23, SE = 0.26, *p* = 0.052), but no significant parietal P3 × RS interaction effect (*β* = 0.01, SE = 0.06, *p* = 0.958).
